# Supplementary material for: Actin–Microtubule Crosstalk Imparts Stiffness to the Contractile Ring in Fission Yeast
Source: Cells. 2023 Mar 16;12(6):917. doi: 10.3390/cells12060917 (PMC10047812; doi:10.3390/cells12060917)
Supplement: Supplementary file 1 [file cells-12-00917-s001.zip › cells-2286900-supplementary.pdf]

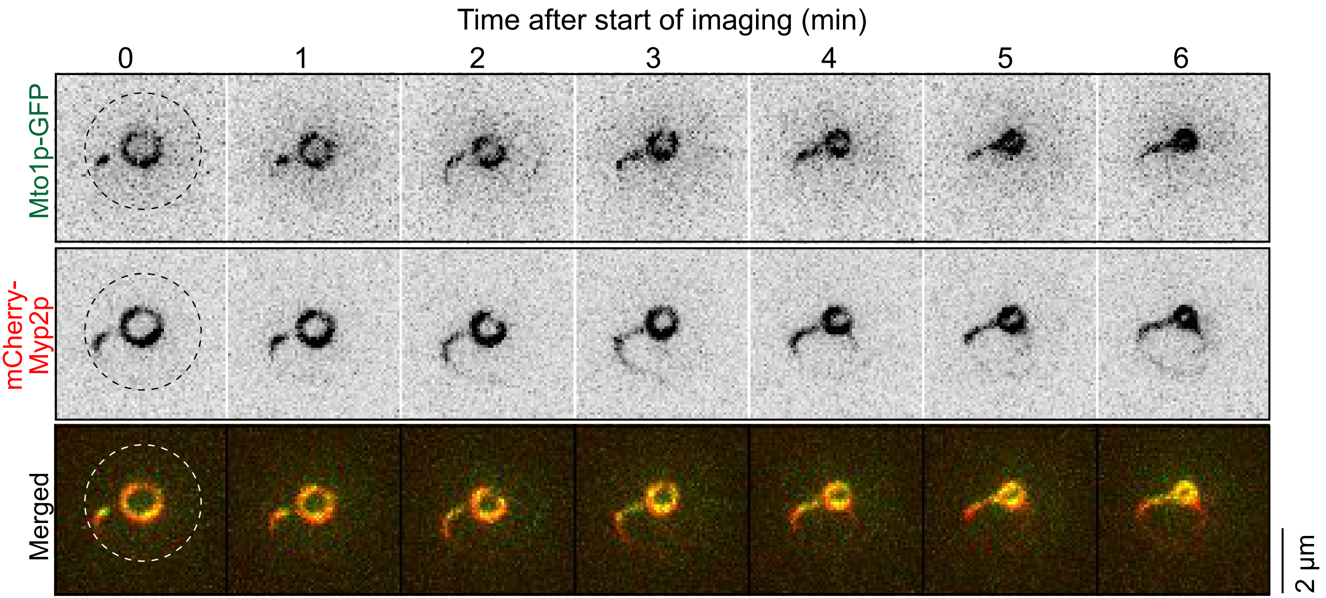

Time after start of imaging (min)
0
1
2
3
4
5
6
Mto1p-GFP
mCherry-Myp2p
Merged
2 µm

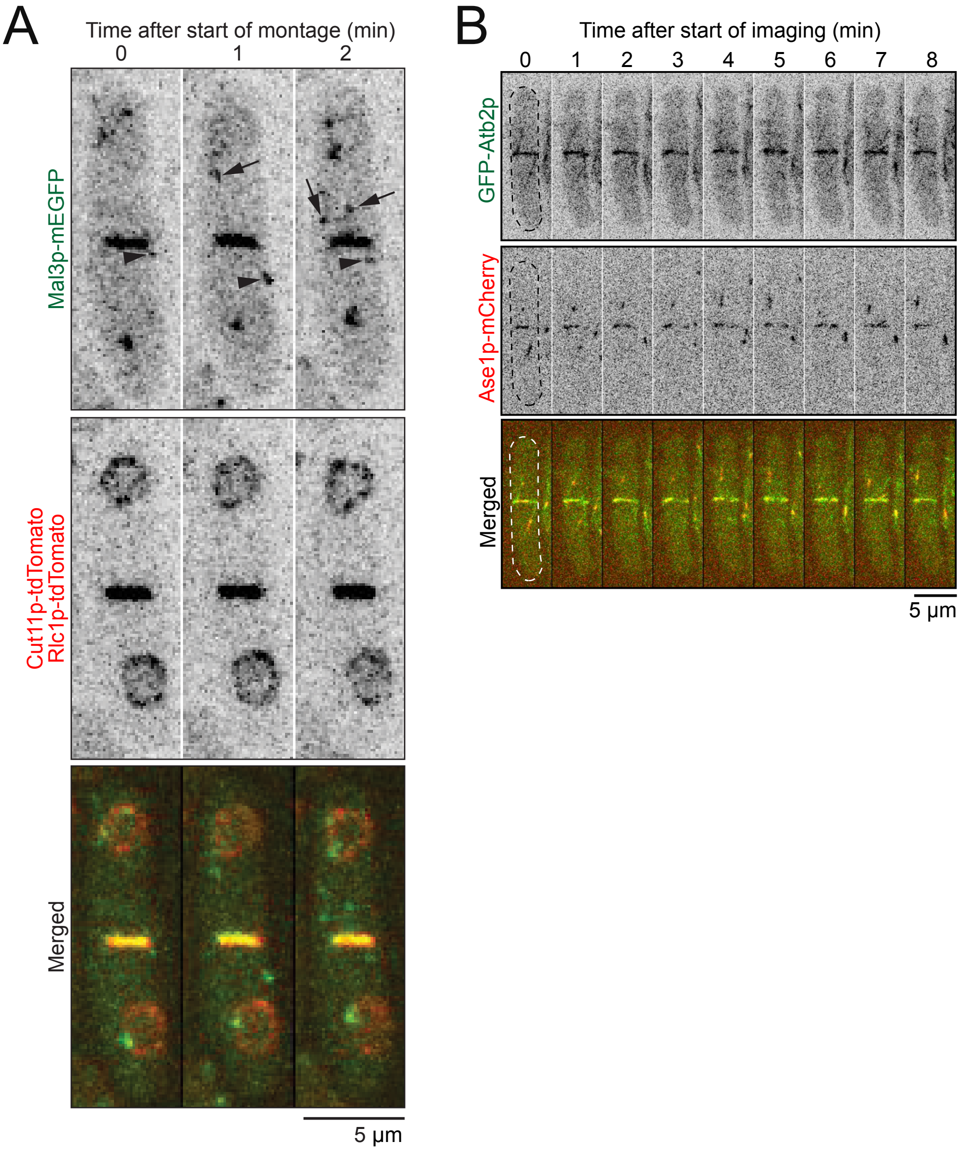
A
Time after start of montage (min)
0
1
2
Mal3p-mEGFP
Cut11p-tdTomato
Rlc1p-tdTomato
Merged
5 µm
B
Time after start of imaging (min)
0
1
2
3
4
5
6
7
8
GFP-Atb2p
Ase1p-mCherry
Merged
5 µm

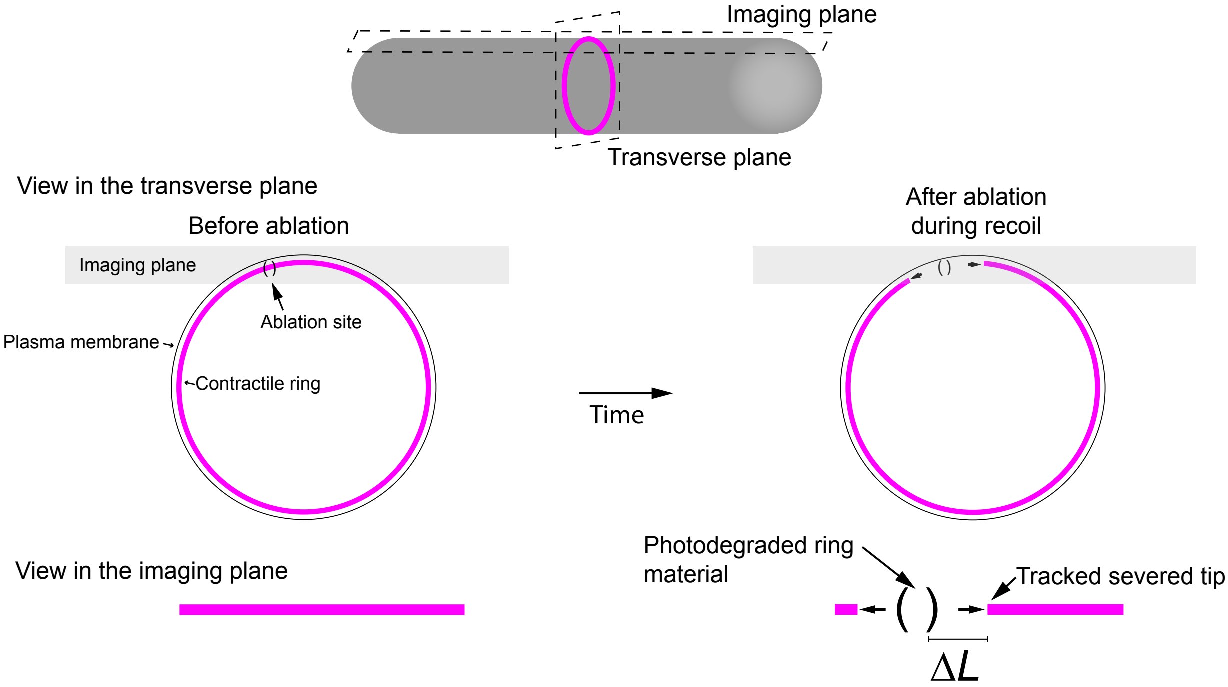
Imaging plane
Transverse plane
View in the transverse plane
Before ablation
Imaging plane
Ablation site
Plasma membrane
Contractile ring
Time
After ablation
during recoil
View in the imaging plane
Photodegraded ring
material
Tracked severed tip
ΔL

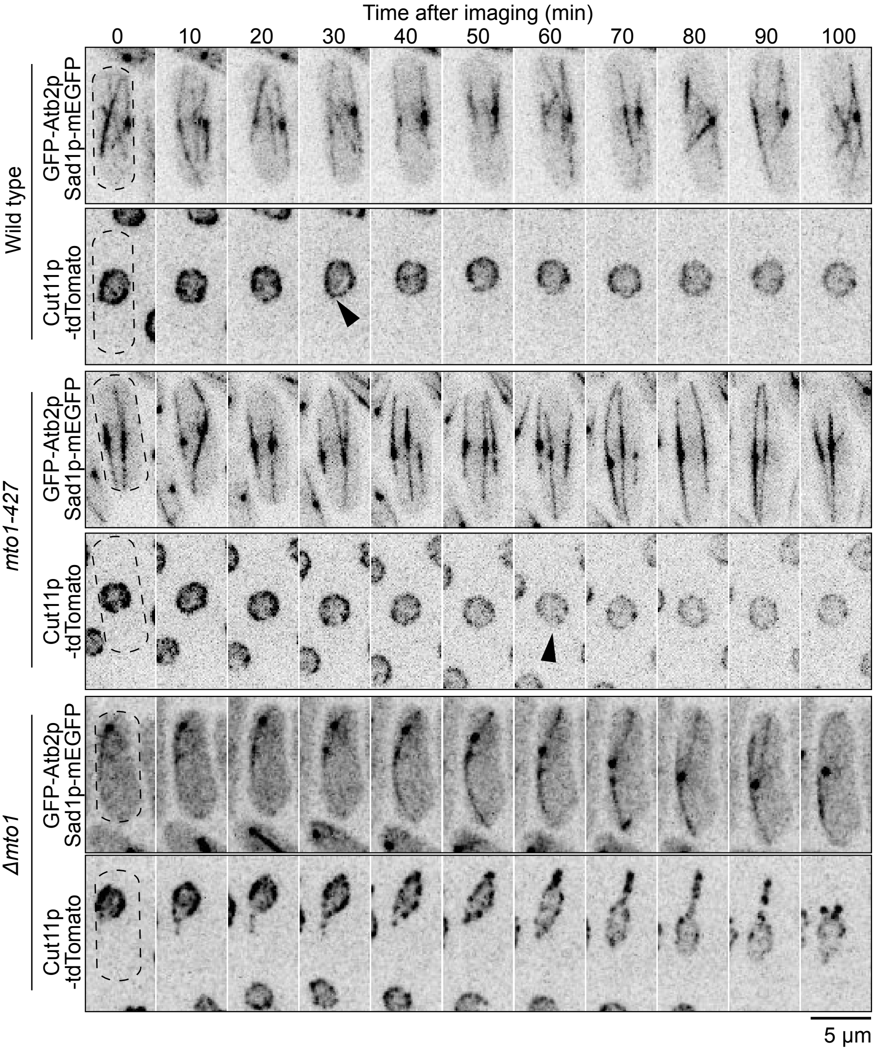
Time after imaging (min)
0
10
20
30
40
50
60
70
80
90
100
Wild type
GFP-Atb2p
Sad1p-mEGFP
Cut11p
-tdTomato
mto1-427
GFP-Atb2p
Sad1p-mEGFP
Cut11p
-tdTomato
Δmto1
GFP-Atb2p
Sad1p-mEGFP
Cut11p
-tdTomato
5 μm

Strain number	Genotype	Reference
CL174	*h+ Pmyp2sh-mEGFP-myp2 ade6-M216 his3-D1 leu1-32 ura4-D18*	Laplante et al., 2015
CL178	*h- kanMX6-Pmyp2sh-mEGFP-myp2 sad1-RFP-kanMX6*	This work.
CL377	*h+ hphMX6-Pnmt81-GFP-atb2*	This work.
CL378	*h+ mto1-GFP-kanMX6 cut12-tdTomato-kanMX6 Pmyp2sh-mCherry-myp2*	This work.
CL380	*hphMX6-Pnmt81-GFP-atb2 Pmyp2sh-mCherry-myp2*	This work.
CL563	*h+ kanMX6Pmyo2sh-mEGFP-myo2 sad1-RFP-kanMX6*	Bellingham-Johnstun et al., 2021
CL584	*h+ cut11-tdTomato-natMX6 hphMX6-Pnmt81-GFP-atb2*	This work.
CL646	*h+ Δmto1::kanMX6 kanMX6-Pmyo2sh-mEGFP-myo2 sad1-RFP-kanMX6*	This work.
CL676	*h+ mal3-mEGFP-kanMX6 cut11-tdTomato-natMX6 rlc1-tdTomato-natMX6*	This work.
CL712	*h+ Δmto1::kanMX6 hphMX6-Pnmt81-GFP-atb2 cut11-tdTomato-natMX6*	This work.
CL719	*h- Δmto1::kanMX6 hphMX6-Pnmt81-GFP-atb2 cut11-tdTomato-natMX6 Sad1-mEGFP-kanMX6*	This work.
CL720	*Δmto1::kanMX6 kanMX6-Pmyo2sh-mEGFP-myo2 cut11-tdTomato-natMX6 sad1-RFP-kanMX6*	This work.
CL721	*h- kanMX6-Pmyo2sh-mEGFP-myo2 cut11-tdTomato-natMX6 sad1-RFP-kanMX6*	This work.
CL739	*h- mto1-427 kanMX6-Pnmt81-GFP-atb2 sad1-dsRed-LEU2 ade6-216 leu1-32 ura4-D18*	Gift from Sawin lab.
CL745	*h+ mto1-427 kanMX6-Pmyo2sh-mEGFP-myo2 sad1-RFP-kanMX6*	Gift from Pollard lab.
CL751	*h- cut11-tdTomato-natMX6 hphMX6-Pnmt81-GFP-atb2 sad1-mEGFP-kanMX6*	This work.
CL754	*h+ mto1-427 cut11-tdTomato-natMX6 hphMX6-nmt81-GFP-atb2 sad1-mEGFP-kanMX6*	This work.
CL756	*h- mto1-427 kanMX6-Pmyo2sh-mEGFP-myo2 cut11-tdTomato-natMX6 sad1-RFP-kanMX6*	This work.
CL972	*mto1-427 kanMX6-Pmyp2sh-mEGFP-myp2 ade6-M216 leu1-32 ura4-D18*	This work.
CL973	*Δmto1::kanMX6 kanMX6-Pmyp2sh-mEGFP-myp2 ade6-21X leu1-32 ura4-D18*	This work.
CL997	*mto1-GFP-kanMX6 Pnmt41-mCherry-CHD-leu1+*	This work.
CL1003	*ase1-mCherry-kanMX6 hphMX6-Pnmt81:GFP-atb2*	This work.
CL1007	*Δase1::natMX6 hphMX6-Pnmt81:GFP-atb2*	This work.
CL1009	*Pmyp2sh-mEGFP-myp2-Tmyp2-leu1+ kanMX6-Pmyp2sh-mEGFP-myp2 mto1-mCherry-kanMX6*	This work.
CL1010	*Pmyp2sh-mEGFP-myp2 mto1-mCherry-kanMX6 ade6-M21X leu1-32 ura4-D18*	This work.
CL1011	*kanMX6-Pmyp2sh-mEGFP-myp2 ase1-mCherry-kanMX6 ade6-M21X leu1-32 ura4-D18*	This work.

Figure S1. Mto1p co-enrich with Myp2p without perfect colocalization. Timelapse micrographs of a cell expressing Mto1p-GFP and mCherry-Myp2p imaged in a yeast motel. Dashed circle, cell outline. Micrographs are inverted grayscale LUT, except merged color images.

Figure S2. Ase1p accumulates with microtubules in the contractile ring and Mal3p labels the +tip/distal ends of the IPAA microtubules. (**A)** Timelapse micrographs of cell expressing Mal3p-mEGFP, Cut11p-tdTomato and Rlc1p-tdTomato. Arrowheads point to the tip of a dynamic IPAA microtubule +tip. Arrows point to other IPAA microtubule +tips in the cell. (**B)** Timelapse micrographs of cell expressing GFP-Atb2p and Ase1p-mCherry. Dashed outlines, cells. Micrographs are shown as inverted grayscale LUT except merged color images.

Figure S3. Probing the mechanical properties of the contractile ring by laser ablation. Diagram of the method of laser ablation of contractile rings (modified from [15]).

Figure S4. Interphase nucleus in Δ*mto1* cells show exaggerated motions and a lemon shape. Timelapse micrographs of cells expressing GFP-Atb2p, Sad1p-mEGFP, and Cut11p-tdTomato in wild-type, Δ*mto1*, and *mto1-427* cells to show differences in nuclear shape and movements. Arrowheads, typical transient deformations in nuclear envelope caused by forces exerted by microtubules. Dashed outline, cells. All micrographs are shown as inverted grayscale LUT.

Table S1. Table of strains.
